# Adverse childhood experiences, brain function, and psychiatric diagnoses in a large adult clinical cohort

**DOI:** 10.3389/fpsyt.2024.1401745

**Published:** 2024-10-14

**Authors:** David B. Keator, Frank Salgado, Caroline Madigan, Sydnyy Murray, Stephanie Norris, Daniel Amen

**Affiliations:** ^1^ Research Department, Change Your Brain Change Your Life Foundation, Costa Mesa, CA, United States; ^2^ Research Department, Amen Clinics, Costa Mesa, CA, United States; ^3^ Department of Psychiatry and Human Behavior, School of Medicine, University of California, Irvine, Irvine, CA, United States; ^4^ Chicago Medical School, North Chicago, IL, United States; ^5^ Department of Psychology, University of California, Davis, Davis, CA, United States

**Keywords:** adverse childhood experiences (ACEs), brain function, neuroimaging, SPECT, psychiatric disorders

## Abstract

**Introduction:**

Adverse childhood experiences (ACEs) are linked to higher rates of psychiatric disorders in adults. Previous neuroimaging studies with small samples have shown associations between ACEs and alterations in brain volume, connectivity, and blood flow. However, no study has explored these associations in a large clinical population to identify brain regions that may mediate the relationship between ACEs and psychiatric diagnoses. This study aims to evaluate how patient-reported ACEs are associated with brain function in adults, across diagnoses.

**Methods:**

We analyzed 7,275 adults using HMPAO SPECT scans at rest and during a continuous performance task (CPT). We assessed the impact of ACEs on brain function across psychiatric diagnoses and performed mediation analyses where brain functional regions of interest acted as mediators between patient-reported ACEs and specific psychiatric diagnoses. We further evaluated the risk of being diagnosed with specific classes of mental illnesses as a function of increasing ACEs and identified which specific ACE questions were statistically related to each diagnosis in this cohort.

**Results:**

Increased ACEs were associated with higher activity in cognitive control and default mode networks and decreased activity in the dorsal striatum and cerebellum. Higher ACEs increased the risk of anxiety-related disorders, substance abuse, and depression. Several brain regions were identified as potential mediators between ACEs and adult psychiatric diagnoses.

**Discussion:**

This study, utilizing a large clinical cohort, provides new insights into the neurobiological mechanisms linking ACEs to adult psychiatric conditions. The findings suggest that specific brain regions mediate the effects of ACEs on the risk of developing mental health disorders, highlighting potential targets for therapeutic interventions.

## 1 Introduction

Adverse childhood experiences (ACEs) include abuse, neglect, and household instability, and are associated with both physical and mental health problems in adulthood. More than half of all adults report at least one ACE, with a quarter of all adults experiencing two or more (1). A large, cross-sectional study of more than 11,000 adults found that there was a dose-dependent association between cumulative ACE scores and psychiatric diagnoses in adults (2). These associations highlight the importance of studying the effects of ACEs on all aspects of health, especially mental health. To date, ACE screening has been completed through questionnaires that assess an individual’s exposure to the 10 ACEs: physical, sexual, and emotional abuse; emotional and physical neglect; domestic violence; and parental substance use, mental illness, separation, and incarceration. Previous research has demonstrated that higher ACE scores are associated with altered adult brain function, which may mediate the psychiatric diagnoses that are prevalent in groups exposed to ACEs (3). Devi et al. found higher rates of psychiatric diagnoses, including mood disorders, schizophrenia, adjustment disorders, and anxiety disorders, in adults with childhood trauma as compared to the general population. Another study by Baldwin et al. found that ACE scores are good predictors of differences in health between groups that did and did not have ACEs, which identified targets for intervention on a broad scale (4). Additional studies have demonstrated the increased risk of mental health disorders with early life adversity such as personality disorders, substance disorders, behavioral disorders, and sleep disorders (5–7). Furthermore, studies have found that childhood maltreatment leads to increased and overgeneralized fear responses in female children, which may predispose them to psychopathology in adulthood (8).

Functional neuroimaging offers a potential bridge between population-level ACE associations and individual mental health diagnoses. ACEs and childhood trauma have been linked to alterations in brain structure, function, and connectivity. It has been hypothesized that ACEs change the connectivity within the brain in a way that results in functional changes leading to later mental health conditions. To date, much of the functional neuroimaging research has been limited by examining single diagnoses, like depression or posttraumatic stress disorder (PTSD) (9). Xie et al. (10) found that reduced right hypothalamic volumes as a reaction to childhood stress may contribute to the development of PTSD in adult trauma survivors. Additionally, neuroimaging work has shown that larger amygdala volumes, as assessed by magnetic resonance imaging (MRI), are correlated with increased risk exposure during childhood (11). A meta-analysis evaluating functional MRI in 34 studies of adults reporting ACEs compared to controls found increased activation in the right amygdala and decreased activation in the middle frontal gyrus (12). Furthermore, in 23 studies of mixed adversity, they found greater activation in the right amygdala, precuneus, and superior frontal gyrus (12). Using positron emission tomography (PET), an imaging modality used to study *in situ* blood flow, Schmahl et al. studied 20 women with a history of childhood physical and sexual abuse and found that memories of trauma were associated with increased blood flow in the right dorsolateral prefrontal cortex (DLPFC), right anterior cingulate, and left orbitofrontal cortex, while they found decreased blood flow in the left DLPFC in women without a borderline personality disorder, suggesting that these functional alterations are associated with early childhood trauma (13). In another PET study using a ligand measuring dopamine, Egerton et al. evaluated 47 young adults compared to 20 healthy volunteers and found that severe physical and sexual abuse and unstable families in childhood were associated with elevated dopamine in the striatum in adulthood, providing evidence that childhood adversity is linked to elevated striatal dopamine function in adulthood (14). Although each of these studies provides evidence linking ACEs to the adult brain, each is limited by small sample sizes and a narrow scope of psychiatric conditions, which limit their generalizability and application in a clinical setting; yet, these studies support additional scientific research to connect ACEs and mental health disorders through functional neuroimaging assessments.

This study aims to understand the relationship between ACEs, psychiatric disorders, and adult brain function in the largest clinical cohort we are aware of to gain insight into the common brain functional patterns associated with increasing ACEs in adults and their risk of being diagnosed with specific mental health illnesses. We hypothesize that psychiatric disorders such as substance abuse/dependence, PTSD, personality disorders, anxiety disorders, and sleep disorders will be associated with higher ACE scores. Furthermore, we expect higher ACE scores to be associated with altered function in areas of the inferior, middle, and superior frontal gyrus, DLPFC, precuneus, anterior cingulate, amygdala, and striatum.

To understand brain functional relationships with ACEs in this clinical cohort, we utilized available single-photon computed tomography (SPECT) data, an imaging technique for measuring perfusion and metabolic status in the brain (15–17). We evaluated brain SPECT in the same patients at rest and during an attention task to understand differences in the associations between increasing ACEs and adult brain function during attentional demands versus at rest, an important distinction for psychiatric conditions such as attention deficit hyperactivity disorder (ADHD) (18, 19), major depressive disorder (MDD) (20, 21), and PTSD (22, 23), among others. Next, we evaluated the risk of being diagnosed with adult psychiatric conditions as a function of increasing patient-reported ACEs and, through mediation analyses, evaluated which brain functional associations serve as mediators between ACEs and each significant psychiatric condition to better inform treating physicians about the potential impact of ACEs on their adult patient’s brain health.

## 2 Materials and methods

The clinical dataset used in this study consisted of 7,275 patients (**Table 1**) who were evaluated at the 11 Amen Clinics Inc. (ACI) mental health facilities over approximately 2 years in which the ACEs 10-question assessment was acquired at intake (24). Patients were excluded if they had diagnoses of dementia, mild cognitive impairment, epilepsy, brain lesions, tumors, or brain resection (see **Supplementary S1** for frequency of diagnoses and S5 for psychiatric medications reported at intake). All patients in the study gave informed consent to have their anonymous data used in future research at the time of their initial visit to the clinic. Each patient participated in two brain SPECT scans as part of their standard intake, one at rest and another while performing the Conners Continuous Performance Test (CPT, Multi-Health Systems, Toronto, Ontario) (25–27). Diagnostic data used in this study were assigned by the ACI treating physician based on all the available clinical data, including a detailed clinical history, DSM IV/V checklists, neuropsychological assessments, and radiological assessments of the brain SPECT scans.

**Table 1:** Patient sample characteristics including sample size, average age ± standard deviation (std) in years, number of male and female patients using sex at birth, race and ethnicity as a percentage of the sample, average number of Axis 1 diagnoses ± std, average ACE total scores ± std, and the distribution of ACE total scores in the sample.

Sample	Age (years)	Sex at Birth	Race and ethnicity	Number of Axis 1 Diagnoses	ACE Total Score	ACE Total Score Distribution
7,275	40.9 ± 16.3 (min = 18; max = 95)	3,521 men 3,754 women	African American: 1.6% Arab/Middle Eastern: 0.3% Asian: 2.9% Caucasian: 75.6% Hispanic/Latino: 1.2% Native American/Inuit: 0.2% Pacific Islander: 0.1% Multi-Ethnic: 0.8% Other: 1.4% Unknown/Declined: 15.8%	2.4 ± 1.6	2.4 ± 2.3	0 = 26.8% 1 = 17.5% 2 = 13.8% 3 = 12.3% 4 = 9.9% 5 = 7.3% 6 = 5.5% 7 = 3.2% 8 = 2.0% 9 = 1.2% 10 = 0.5%

SPECT scans were acquired using InterMedical MultiCam 3000eco 3-head gamma cameras (Intermedical Medizintechnik GmbH, Lubbecke, D-32312, Germany). For each procedure, an age- and weight-appropriate dose of 99mTc–hexamethylpropyleneamine oxime (HMPAO) was administered intravenously at rest and while performing the CPT, a visual task used for the evaluation of attention and response inhibition (25–27). For the resting scans, patients were injected while they sat in a dimly lit room with their eyes open. For the CPT scans, patients were injected 3 min after starting the task. Approximately 15 min after the injection, the patients were scanned. CPT scans were collected on average 1.8 ± 2.7 days after the resting scans. Data acquisition yielded 120 images per scan, with each image separated by 3°, spanning 360°. A low-pass filter was applied with a high cutoff, and Chang attenuation correction was performed (28, 29). The resulting reconstructed image matrices were 128 × 128 × 78 with voxel sizes of 2.5 mm^3^.

For voxel-based analyses, images were aligned to the Montreal Neurological Institute (MNI) space with the Advanced Normalization Tools [ANTs version 2.2.0 (30); RRID: SCR_004757] using a SPECT template, resulting in an image matrix size of 79 × 96 × 68 with isotropic voxel sizes of 2.0 mm^3^. SPECT images were scaled to the within-scan maximum voxel, and noise outside of the brain was removed using 50% of the maximum threshold prior to registration. After the thresholded images were aligned to the MNI space and the transformation was applied to the un-thresholded images, a brain mask derived from the MNI 152 template (31, 32) was used to remove noise outside the brain from the un-thresholded images for use in the statistical models. Registered SPECT scans were visually checked for the absence of severe anatomical abnormalities and/or proper registration to the MNI space. All subsequent voxel-based linear models were constructed using the SPM12^1^ Statistical Parametric Mapping tool (RRID: SCR_007037) (33). A voxel-based one-sample *t*-test model was used to compare the ACE total score with cerebral perfusion using proportional scaling to account for differences in global SPECT signal. To account for differences in ventricular off-target noise, we followed a procedure similar to CompCor (34), adding the mean and maximum activity in the ventricles as nuisance regressors to the SPM models. Because the data used in this study are from a clinical sample of patients collected at multiple imaging facilities and with varying diagnoses and comorbidities, both the acquisition site and number of axis 1 psychiatric diagnoses (35) were included as nuisance covariates (see **Supplementary S1** for frequency of diagnostic classes in the sample) along with age and patient-reported sex at birth.

Each patient was asked to complete the ACE assessment, consisting of 10 questions listed in **Table 2**. To evaluate the risk of being diagnosed with a specific class of mental health condition as a function of increasing the ACE total score and to associate individual question responses with each significant diagnosis, linear binomial regression models were used in the *R* ^2^ statistical analysis software (RStudio version 2023.06.0 + 421; RRID: SCR_001905), including age, patient-reported sex at birth, and data acquisition site as nuisance covariates.

**Table 2:** Adverse childhood experiences (ACE) assessment questions.

ACE_Q_1	Before your 18th birthday, did a parent or other adult in the household often or very often swear at you, insult you, put you down, or humiliate you? Or act in a way that made you afraid that you might be physically hurt?
ACE_Q_2	Before your 18th birthday, did a parent or other adult in the household often or very often push, grab, slap, or throw something at you? Or, ever hit you so hard that you had marks or were injured?
ACE_Q_3	Before your 18th birthday, did an adult or person at least five years older than you ever touch or fondle you, or have you touch their body in a sexual way? Or attempted or actually had oral, anal, or vaginal intercourse with you?
ACE_Q_4	Before your 18th birthday, did you often or very often feel that no one in your family loved you or thought you were important or special? Or that your family did not look out for each other, feel close to each other, or support each other?
ACE_Q_5	Before your 18th birthday, did you often or very often feel that you did not have enough to eat, had to wear dirty clothes, and had no one to protect you? Or that your parents were too drunk or high to take care of you or take you to the doctor if you needed it?
ACE_Q_6	Before your 18th birthday, was a biological parent ever lost to you through divorce, abandonment, or other reasons?
ACE_Q_7	Before your 18th birthday, was your mother or stepmother: often or very often pushed, grabbed, slapped, or had something thrown at her? Or, sometimes, often, or very often kicked, bitten, hit with a fist, or something hard? Or, ever repeatedly hit over at least a few minutes or threatened with a gun or knife?
ACE_Q_8	Before your 18th birthday, did you live with anyone who was a problem drinker or alcoholic, or who used street drugs?
ACE_Q_9	Before your 18th birthday, was a household member depressed or mentally ill, or did a household member attempt suicide?
ACE_Q_10	Before your 18th birthday, did a household member go to prison?

To understand whether the significant brain functional associations with ACEs serve as mediators between ACEs and adult psychiatric diagnoses, we used the multilevel mediation and moderation (M3) toolbox (36–38). The mediator variable (*m*) was the mean voxel value across 5^3^ mm boxed regions of interest (ROIs) centered on the coordinates reported in **Tables 3** and **4**. The mean ROI data for each patient in the study were sampled from the SPECT data used in the SPM voxel-based models with the MarsBar toolbox (39). Path (b) in **Figure 3** represents the association between mean brain function in each ROI and the diagnostic class (*y*). Covariates for the mediator variables included age, sex at birth, site, and number of Axis 1 diagnoses. The path (ab) represents the mediating effect of brain function between the ACE total score and the diagnostic class. The direct path between ACEs and the diagnostic class is represented by path (c′), after controlling for the mediator variable (*m*). For a full mediation, we required the probabilities of the coefficients to satisfy the following criteria: a(*p*) < 0.05, b(*p*) < 0.05, ab(*p*) < 0.05, and c′(*p*) > 0.05, indicating that the path through the brain function mediator variable is significant, whereas the direct path (c′) after controlling for the mediator, is not. For partial mediation, we required the following: a(*p*) < 0.05, b(*p*)< 0.05, and ab(*p*) < 0.05, and allow c′(*p*) < 0.05, but that a(*p*), b(*p*), and ab(*p*) are all less than (i.e., more significant) than the c′(*p*) relationship, indicating that the path through the brain function mediator variable is more statistically significant than the direct path (c′) after controlling for the mediator. We then interpreted the output of the mediation models with respect to full and partial mediation (see Section 3.3).

**Table 3:** Statistically significant [*t*(1,7268) = 2.45, *p* < 0.05 FDR] regions of association with higher ACE scores in the CPT condition after co-varying for the nuisance effects of age, sex at birth, location/site, and number of axis 1 diagnoses.

Coordinates (*x*,*y*,*z*)	Region	*t* (1,7268)	Direction	Cohen’s *D*
Parietal				
−40 −56 40	Angular Gyrus L (BA 39)	5.65^*^	Positive	0.13
40 −54 40	Angular Gyrus R (BA 39)	5.77^*^	Positive	0.13
Sub-cortical				
0 −20 −2	Thalamus Medial Dorsal	4.75^*^	Positive	0.11
26 12 −8	Putamen R – Lentiform Nucleus	4.74^*^	Negative	0.11
−14 16 −2	Caudate L	3.86	Negative	0.09
12 22 −6	Caudate R	3.57	Negative	0.08
−28 10 −8	Putamen L	5.06^*^	Negative	0.12
−32 6 16	Insula L	5.30^*^	Negative	0.13
38 −20 14	Insula R	3.81	Positive	0.09
−20 −40 −12	Parahippocampal L (BA 36)	3.78	Positive	0.09
18 −34 −6	Parahippocampal R (BA 30)	4.50	Positive	0.11
−10 −24 14	Thalamus Pulvinar L	4.05	Negative	0.10
Occipital				
2 −90 −2	Lingual Gyrus (BA 18)	6.03^*^	Positive	0.14
−34 −84 12	Occipital Mid. L (BA 19)	4.60^*^	Positive	0.11
32 −74 12	Occipital Mid. R (BA 19)	3.20	Positive	0.11
Cerebellum				
28 −54 −50	Cerebellum R	5.14^*^	Negative	0.12
−26 −62 −50	Cerebellum L	3.79	Negative	0.09
Cingulate				
−2 34 22	Ant. Cingulate Sup. L (BA32)	3.59	Positive	0.08
0 −42 36	Post. Cingulate (BA 31)	5.01	Positive	0.12
2 6 −8	Ant. Cingulate Ventral	5.01	Positive	0.12
Temporal				
54 −50 18	Temporal Sup R (BA 22)	5.12^*^	Positive	0.12
−58 −30 16	Temporal Sup L (BA 22)	4.67^*^	Positive	0.11
−58 −20 −20	Temporal Inf L (BA 20)	3.36	Positive	0.08
−62 −22 −14	Temporal Mid. L (BA 21)	3.52	Positive	0.08
60 −6 −18	Temporal Mid. R (BA 21)	3.89	Positive	0.09
42 −46 −10	Fusiform R (BA 37)	4.13	Negative	0.09
−30 −58 −10	Fusiform L (BA 37)	3.83	Negative	0.09
Frontal				
−2 60 14	Medial Frontal (BA 10)	5.19^*^	Positive	0.12
4 40 −24	Medial Frontal (BA 11)	4.22	Positive	0.10
−16 44 10	Medial Frontal L (BA 9)	4.42	Negative	0.10
18 54 2	Medial Frontal R (BA 10)	4.12	Negative	0.10
18 24 −22	Inferior Frontal R (BA 47)	6.19^*^	Negative	0.15
−20 24 −22	Inferior Frontal L (BA 47)	4.20	Negative	0.10
Brainstem				
2 −32 −38	Pons	4.57^*^	Negative	0.10

The table includes MNI-space coordinates in millimeters from the origin (coordinate), brain region (Region), *t*-score (*t*), *p*-value (*p*-value) corresponding to the *t*-score, direction of association, either positive or negative (Direction), and the Cohen’s *D* effect size (Cohen’s *D*). Asterisks in the *t*-score (*t*) column indicate differences that survive a more stringent family-wise error correction at *p* < 0.05 [*t*(1,7268) = 4.61, *p* < 0.05 FWE].

**Table 4:** Statistically significant [*t*(1,7268) = 2.45, *p* < 0.05 FDR] regions of association with higher ACE scores during the rest condition after co-varying for the nuisance effects of age, biological sex, location, and number of axis 1 diagnoses.

Coordinates (*x*,*y*,*z*)	Region	*t* (1,7268)	Direction	Cohen’s *D*
Parietal				
40 −54 42	Parietal Inferior R (BA 40)	4.59^*^	Positive	0.11
−26 −60 50	Parietal Superior L (BA 7)	3.56	Positive	0.08
42 −52 40	Angular Gyrus R (BA 39)	4.12	Positive	0.10
−16 −46 44	Precuneus L (BA 7)	3.86	Negative	0.09
18 −44 44	Precuneus R (BA 7)	4.02	Negative	0.09
Sub-cortical				
44 −22 14	Insula R	4.40	Positive	0.10
−34 −4 16	Insula L	3.54	Negative	0.08
12 −14 8	Thalamus—Medial Dorsal R	4.25	Negative	0.10
14 −18 14	Thalamus—Lateral Dorsal R	4.58^*^	Negative	0.11
−18 −24 8	Thalamus—Pulvinar L	4.31	Negative	0.10
−18 1 −12 −20 16 −4	Putamen L	5.66^*^ 4.72^*^	Negative	0.13 0.11
20 14 −12	Putamen—Lentiform R	4.89^*^	Negative	0.11
38 10 14	Insula R (BA 13)	3.09	Negative	0.07
−34 −4 16	Insula L (BA 13)	3.54	Negative	0.08
36 −42 −12	Parahippocampal R (BA 37)	3.72	Negative	0.09
Occipital				
2 −88 −8	Lingual (BA 18)	4.93^*^	Positive	0.12
Cerebellum				
20 −60 −50	Cerebellum R	5.27^*^	Negative	0.12
−22 −60 −48	Cerebellum L	3.95	Negative	0.09
Cingulate				
0 30 26	Ant. Cingulate Sup	4.30	Positive	0.10
−8 −14 34	Mid. Cingulate	2.93	Positive	0.07
−4 −60 8 −4 −46 30	Posterior Cingulate (BA30)	4.56 4.24	Positive	0.11 0.10
Temporal				
−34 −78 24	Temporal Mid L (BA 21)	5.00^*^	Positive	0.12
40 −70 26	Temporal Mid R (BA 21)	4.03	Positive	0.09
46 −24 10	Transverse Temporal (BA41) R	4.72^*^	Positive	0.11
−50 −26 10	Transverse Temporal (BA41) L	4.26^*^	Positive	0.10
Frontal				
0 58 16	Medial Frontal (BA 9)	4.00	Positive	0.09
0 42 −22	Medial Frontal (BA11)	3.61	Positive	0.08
−16 26 −24	Inferior Frontal (BA 47) L	4.34	Negative	0.10
16 26 −24	Inferior Frontal (BA 47) R	4.06	Negative	0.10
Brainstem				
6 −28 −32	Pons	4.12	Negative	0.10

The table includes MNI-space coordinates in millimeters from the origin (coordinate), brain region (Region), *t*-score (*t*), the direction of the association, either positive or negative (Direction), and the Cohen’s *D* effect size (Cohen’s *D*). Asterisks in the *t*-score (*t*) column indicate differences that survive a more stringent family-wise error correction at *p* < 0.05 [*t*(1,7268) = 4.61, *p* < 0.05 FWE].

## 3 Results

### 3.1 SPECT associations with increasing ACEs

**Figure 1:**
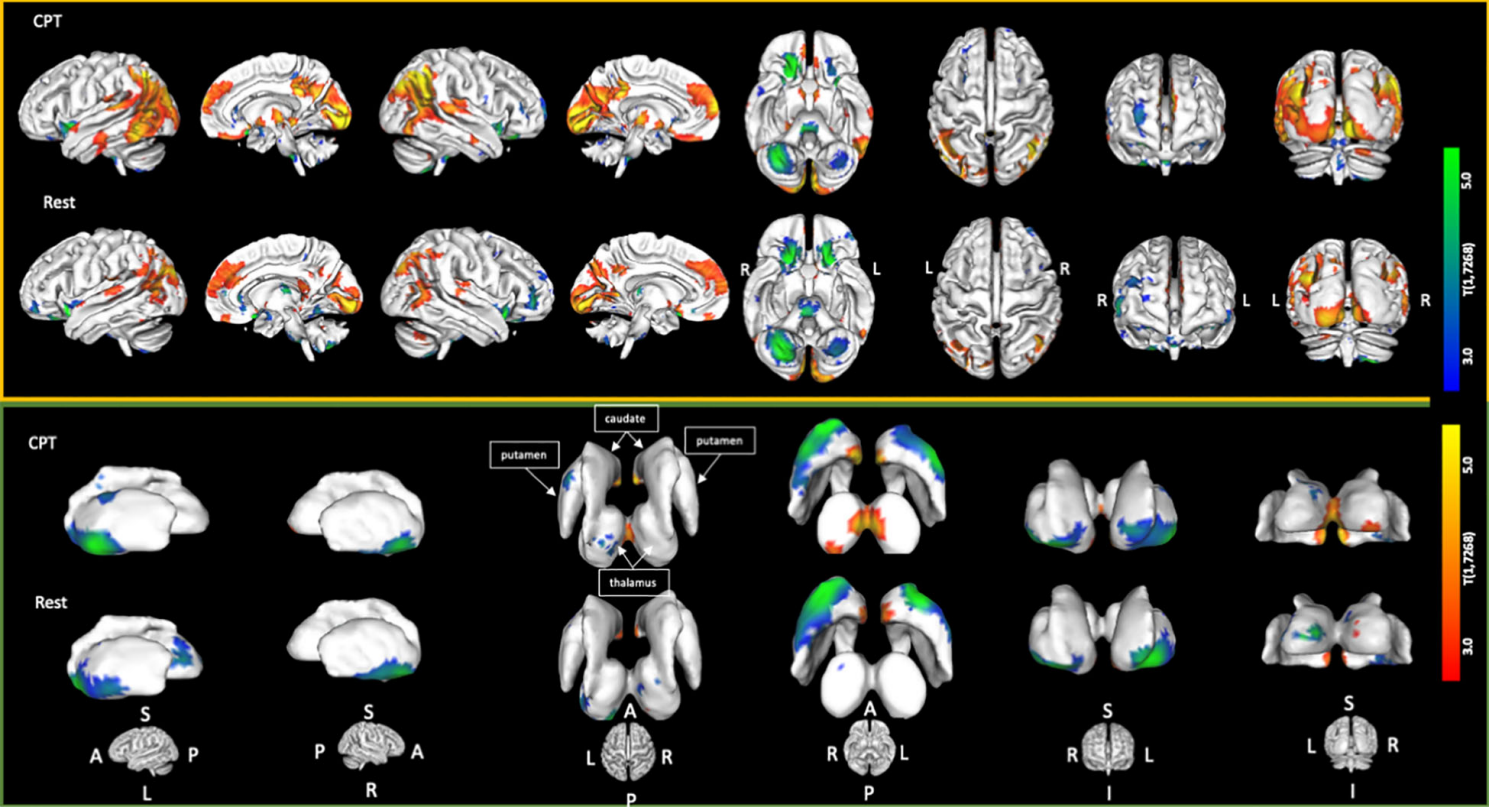
Cortical and sub-cortical areas showing statistically significant [*t*(1,7268) = 2.45; *p* < 0.05 FDR corrected] associations with increasing ACE scores. Areas in red/yellow show regions of higher perfusion with increasing ACE scores. Areas in blue/green show regions of lower perfusion with increasing ACE scores.

**Figure 2:**
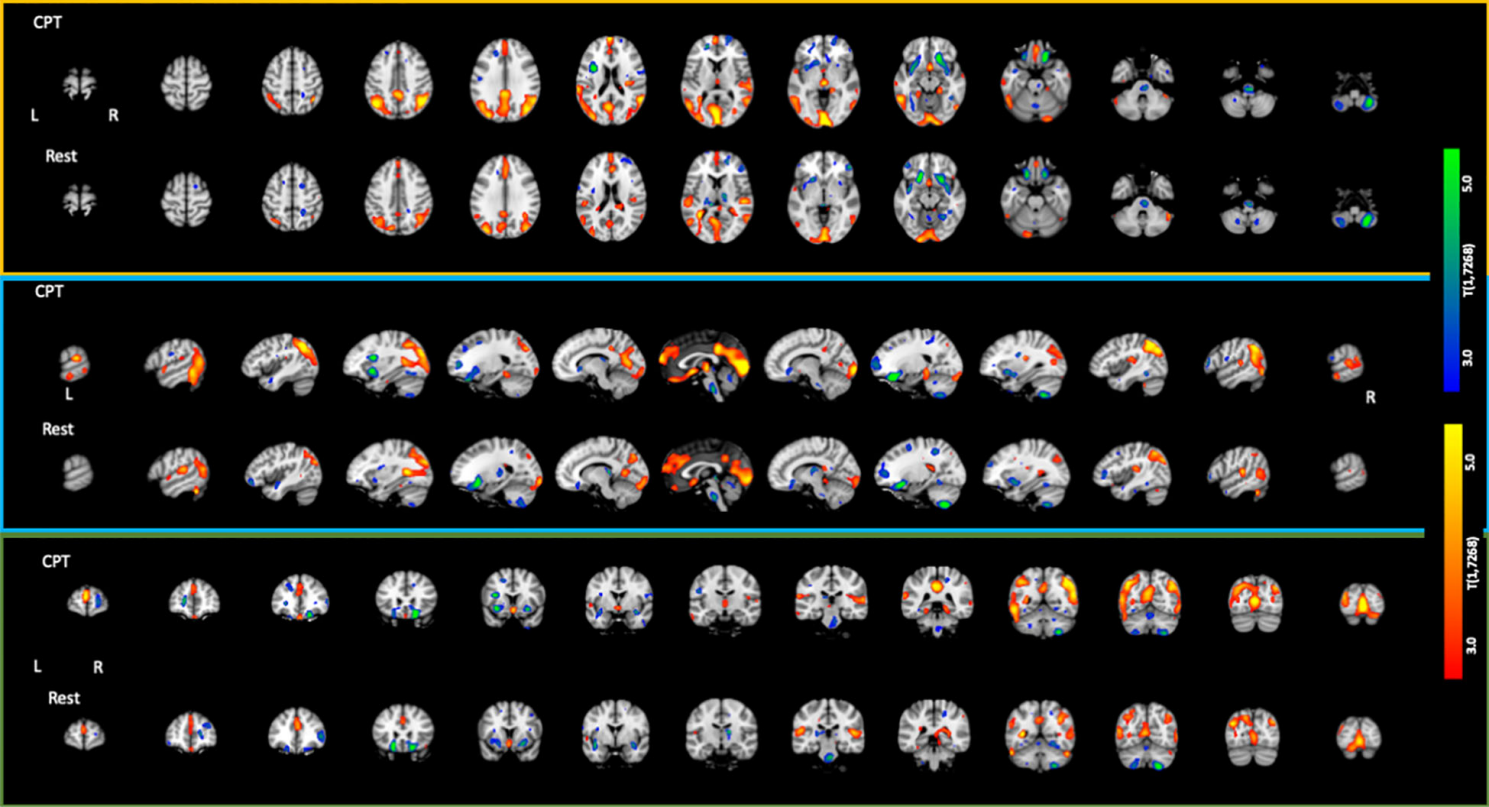
Axial, sagittal, and coronal slices showing statistically significant [*t*(1,7268) = 2.45; *p* < 0.05 FDR corrected] associations with increasing ACE scores. Areas in red/yellow show regions of higher perfusion with increasing ACE scores. Areas in blue/green show regions of lower perfusion with increasing ACE scores.

The CPT SPECT analysis yielded both positive and negative associations with ACE total score (**Table 3**; **Figures 1**, **2**). Significant voxels were evaluated using whole-brain false discovery rate (FDR) multiple comparison corrections [*t*(1,7268) = 2.45, *p* < 0.05 FDR] in SPM12 and reported with MNI-space coordinates and atlas-identified brain regions using the Mango^3^ tool. We found higher perfusion in association with increasing ACEs in areas of the angular gyrus, superior, middle, inferior, and fusiform gyrus of the temporal lobe, parahippocampal gyrus, midline aspects of the medial frontal, medial dorsal nucleus of the thalamus, superior anterior cingulate, ventral anterior cingulate, posterior cingulate, and areas of the visual cortex. We found negative associations in the inferior orbitofrontal cortex, lateral aspects of the medial frontal, caudate, putamen, insula, pulvinar area of the thalamus, cerebellum, and pons region of the brainstem.

The resting condition also showed both positive and negative associations with ACEs, using the same threshold as with the CPT condition (**Table 4**; **Figures 1**, **2**). We found higher perfusion in association with increasing ACEs in areas of the superior and inferior parietal lobe, the angular gyrus, precuneus, middle and transverse temporal lobes, midline aspects of the medial frontal lobe, and areas of the visual cortex, although less widespread than in the CPT condition. In the cingulate, we find positive associations in the superior anterior cingulate, posterior cingulate, and middle cingulate, a region not identified in the CPT condition. We found negative associations in the parahippocampal gyrus, the inferior orbitofrontal cortex, the cerebellum, the caudate, the putamen, the medial and lateral dorsal nuclei and pulvinar of the thalamus, and the pons region of the brainstem. The insula included both positive and negative associations.

In evaluating the relationship between ACEs and the difference between CPT and resting conditions (see **Supplementary Section S.4**), we found only positive associations surviving the FDR threshold, implying that greater activation during the CPT condition over the resting condition is related to increasing ACEs. We found significant clusters in regions of the prefrontal cortex, inferior frontal cortex, insula, portions of the inferior, middle, and superior temporal lobes, anterior, middle, and posterior cingulate, inferior parietal lobe, right cerebellum, visual cortex, caudate, and both medial–dorsal and ventral–lateral thalamus.

### 3.2 Diagnoses

To understand the relationship between increasing ACEs and the risk of specific diagnoses in this sample, we use the diagnoses given at ACI and binomial regression, covarying for age, sex at birth, and site of treatment. The results are shown in **Table 5**. To further evaluate the relationship between the diagnostic classes identified in **Table 5** and specific ACE questions, we regressed each diagnosis against all ACE questions, covarying for age, sex at birth, and site of treatment (see **Supplementary Section S2** for significant ACE question statistics). We then selected ACE questions that were significantly (*p* < 0.05 FDR) related to each diagnostic class. These results identify many diagnostic classes that have been reported in the literature to be associated with ACEs, such as PTSD, anxiety disorders, mood disorders, and a variety of substance abuse-related disorders (see the Discussion section). We found that PTSD was significantly associated with the majority of ACE questions, including those involving psychological abuse, sexual abuse, household dysfunction, violence, and mental illness. Furthermore, we found that substance abuse-related diagnoses (i.e., substance abuse, substance dependence, alcohol-related disorder, and nicotine-related disorder) were all associated with having substance abuse in the household as a child, with a subset also associated with having a mental illness and/or psychological abuse present. The depression-related diagnoses were associated with having psychological abuse and/or mental illness in the household, which is consistent with our clinical expectations. Finally, attention deficit-related disorders were associated with sexual abuse and violence in the household.

**Table 5:** The diagnostic classes significantly (*p* < 0.05 FDR) associated with increasing ACE scores.

Diagnostic Class	*t*(1,7164)	Odds Ratio	*p*-Value (FDR Corrected)	Significant ACE Questions (*p* < 0.05 FDR)
Post-Traumatic Stress Disorder	24.94	1.36	1.01E−135	1,3,4,5,7,9
Anxiety Disorder	10.14	1.14	6.03E−23	9
Brain Trauma	9.27	1.19	2.00E−19	1,4,6
Depression NOS	7.15	1.13	7.39E−12	9
Parasomnia	6.73	1.32	1.10E−10	5
Borderline	6.16	1.28	4.04E−09	
Personality Cluster B	6.03	1.27	7.78E−09	
Attention Deficit Disruptive Behavior	5.97	1.07	9.66E−09	3,7
Attention Deficit Hyperactivity	5.94	1.07	1.02E−08	3,7
Depressive Disorder	5.55	1.06	9.27E−08	4,9
Bipolar Disorder	5.33	1.10	2.90E−07	
Substance Abuse Disorder	5.02	1.09	1.44E−06	8,9
Alcohol-Related Disorder	3.97	1.08	1.84E−04	8
Major Depression	3.66	1.04	5.96E−04	4,9
Mood Disorder NOS	3.32	1.06	1.97E−03	
Primary Sleep Disorder	2.53	1.08	2.09E−02	
Phobias	2.48	1.06	2.17E−02	
Cyclothymic Disorder	2.48	1.12	2.17E−02	
Nicotine-Related Disorders	2.45	1.08	2.24E−02	4,8,9
Substance Dependence	2.35	1.06	2.83E−02	8

The table includes the *t*-score for the ACE total score variable, odds ratio of the exponentiated ACE total score coefficient, uncorrected *p*-values, FDR-corrected *p*-values, and ACE questions associated with each diagnostic class.

### 3.3 ACEs—brain function mediation

**Figure 3:**
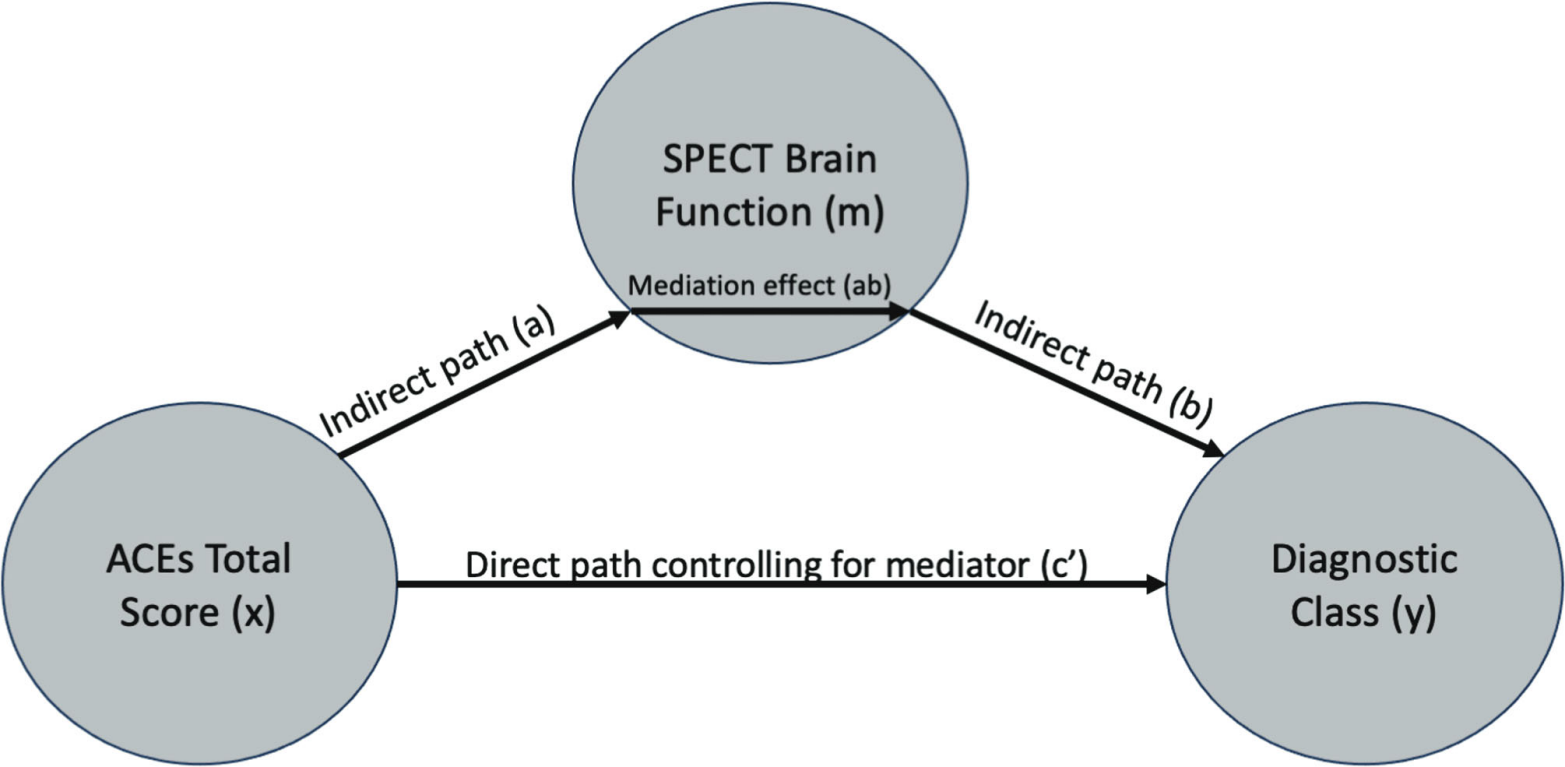
Mediation model path diagram showing each component of the models: ACE total score (*x*), SPECT brain function meditation variable (*m*), and diagnostic outcome variable (*y*).

In Section 3.1, we showed the significant brain function associations with increasing ACEs. In Section 3.2, we related increasing ACEs to the risk of being diagnosed with specific mental health conditions. Our hypothesis is that having ACEs as a child may alter one’s perception of the world, their experiences in it, and brain development/function. Such aberrant brain function, in adulthood, could be a potential mediator between ACEs and mental health diagnoses. Here, we specifically tested these mediation relationships. For each diagnosis in **Table 5** and each significant ACE-SPECT association in **Tables 3** and **4**, we evaluated full and partial mediation effects with SPECT-derived brain function as the mediator. The models were constructed using similar procedures to those described in (36–38). In our models (**Figure 3**), the initial variable was the ACE total score (*x*), with a path between the ACE total score and brain function (a), representing the association between ACEs and SPECT brain function. The FDR-corrected results are shown in **Table 6**, while the complete table including significant results at the uncorrected threshold of *p* < 0.05 is provided in **Supplementary Section S3**.

**Table 6:** Mediation model results by diagnostic class.

Diagnostic Class	Brain Region (Coordinates)	Condition	Mediation Type	a(*t*)	a(*p*)	b(*t*)	b(*p*)	ab(*t*)	ab(*p*)	c'(*t*)	c'(*p*)
Substance Abuse Disorder	Putamen L (−20,16,−4)	Rest	Full	−5.12	**3.10E**−**07**	3.04	**2.37E**−**03**	−2.58	**9.95E**−**03**	1.48	1.38E−01
	Insula L (−32,6,16)	CPT	Full	−5.04	**4.74E**−**07**	3.53	**4.16E**−**04**	−2.85	**4.32E**−**03**	1.51	1.31E−01
	Caudate L (−14,16,−2)	CPT	Full	−5.51	**3.65E**−**08**	2.86	**4.28E**−**03**	−2.51	**1.23E**−**02**	1.49	1.37E−01
Substance Dependence	Medial Frontal (0,58,16)	Rest	Full	4.57	**4.85E**−**06**	−2.81	**4.92E**−**03**	−2.36	**1.85E**−**02**	0.52	6.02E−01
	Medial Frontal (−2,60,14)	CPT	Full	5.05	**4.56E**−**07**	−2.49	**1.29E**−**02**	−2.20	**2.80E**−**02**	0.52	6.05E−01
	Medial Frontal L (−16,44,10)	CPT	Full	−3.64	**2.70E**−**04**	2.80	**5.07E**−**03**	−2.17	**3.00E**−**02**	0.49	6.24E−01
Alcohol-Related Disorder	Parietal Superior L (−26,−60,50)	Rest	Partial	3.49	**4.93E**−**04**	−3.96	**7.60E**−**05**	−2.57	**1.02E**−**02**	2.25	**2.43E**−**02**
	Parahippocampal R (18,−34,−6)	CPT	Full	4.52	**6.24E**−**06**	2.40	**1.62E**−**02**	2.08	**3.73E**−**02**	1.96	5.01E−02
	Insula L (−32,6,16)	CPT	Partial	−5.04	**4.74E**−**07**	2.77	**5.61E**−**03**	−2.39	**1.68E**−**02**	2.25	**2.44E**−**02**
Nicotine-Related Disorder	Pons (2,−32,−38)	CPT	Full	−5.14	**2.86E**−**07**	−2.24	**2.52E**−**02**	2.02	**4.34E**−**02**	0.51	6.10E−01
Mood Disorder NOS	Pons (6,−28,−32)	Rest	Partial	−4.82	**1.47E**−**06**	3.35	**8.13E**−**04**	−2.71	**6.72E**−**03**	2.45	**1.44E**−**02**
	Parahippocampal L (−20,−40,−12)	CPT	Partial	3.49	**4.79E**−**04**	−6.26	**3.96E**−**10**	−3.02	**2.52E**−**03**	2.52	**1.17E**−**02**
	Thalamus Medial Dorsal (0,−20,−2)	CPT	Partial	4.89	**1.04E**−**06**	3.36	**7.85E**−**04**	2.73	**6.34E**−**03**	2.07	**3.89E**−**02**

The table shows the central coordinate of the ROI used to sample SPECT data (Brain Region), condition the result applies to (i.e., Resting or CPT), mediation type (i.e., Full or Partial), *t*-score (1,7207), and FDR-corrected *p*-values for each path in the mediation model. *p*-values for each link in the mediation model that are significant at *p* < 0.05 FDR-corrected are shown in bold.

Overall, we found both full and partial mediation results in resting and CPT SPECT scan ROIs. Looking broadly at the results, we found that the substance abuse disorder diagnosis includes the insula, putamen, and caudate. In comparison, the substance dependence diagnosis yields the strongest results in the medial frontal regions. For alcohol-related disorders, we found that the left superior parietal and left insula are partial mediators, whereas the right parahippocampal gyrus is a full mediator. For nicotine-related disorders, we found that the pons are a significant mediator only in the CPT condition. For mood disorders, we found partial mediators, including the pons at rest, the bilateral parahippocampal gyrus in the CPT condition, and the medial dorsal nucleus region of the thalamus in the CPT condition. There were no significant full or partial mediation results when evaluating the difference between CPT and resting scans.

## 4 Discussion

This study offers significant insights into how ACEs are associated with brain function and psychiatric diagnoses in a large clinical cohort. The findings underscore the profound impact of ACEs on adult mental health, highlighting the need for clinicians to incorporate this knowledge into practice to better diagnose and treat individuals with a history of childhood adversity.

### 4.1 Neuroimaging

In the SPECT neuroimaging analyses, we found a mix of positive and negative associations in the frontal and parietal regions that are part of the cognitive control network. Both resting and CPT scans yielded positive associations in the medial aspects of the frontal lobe (BA 9-11). This may be evidence of difficulty in decision-making due to overactivity at rest and more so in the CPT condition, as a function of higher ACEs. Brodmann areas 9–11, part of the prefrontal cortex, have been associated with a variety of functions including risk and decision-making, learning, planning, focus, reward and conflict, pain, pleasant and unpleasant emotions, anhedonia, and working memory (40–42). Furthermore, in both resting and CPT scans, we found negative associations in the orbital frontal region (BA 47), bilaterally, which may indicate a dulling of emotion recognition. BA 47 is thought to be related to the recognition of emotions such as fear, disgust, and anger (43). In the parietal lobe, we found consistent positive associations in the angular gyrus, on the right side at rest and bilaterally in the CPT condition. The angular gyrus is thought to be a hub of converging multisensory information and has been associated with a variety of functions including conflict resolution, episodic memory, theory of mind, visuospatial retrieval, default mode network, inhibition, and memory retrieval (44).

Broadly, the cognitive control network has been associated with regions in the DLPFC, anterior cingulate, insula, angular gyrus, and parietal cortex (45, 46). Deficits in the cognitive control network have been linked with alcohol use disorder, substance use disorder, bipolar disorder, depression, and PTSD, among others (47–49). In our results, we found significant associations in areas of cognitive control in both resting and CPT conditions, while also showing that substance abuse, substance dependence, alcohol abuse, nicotine-related disorders, PTSD, and depression-related diagnoses were all significantly associated with higher ACEs. Furthermore, we found that regions of the cognitive control network in both resting and CPT conditions were mediators between ACEs and substance abuse, substance dependence, and alcohol-related disorders at the uncorrected significance level (see **Supplementary Table S3**). One interpretation is that having more ACEs as a child is related to altered brain function as an adult in areas of the cognitive control network that mediate the association between ACEs and these specific diagnoses. Because each of these disorders is related to drugs of abuse, and because we know that drug abuse damages the brain (50–54), an alternative interpretation is that having more ACEs as a child is associated with an increased risk of abusing drugs, which results in aberrant functioning in cognitive control networks. There is support in the literature for this alternative interpretation by studies showing that ACEs are associated with increased drug abuse (55–57). Interestingly, we found that substance abuse, substance dependence, and alcohol-related disorders were all associated with ACE question 8, which asks whether the individual grew up in a household with drugs of abuse. These data suggest that having substance abuse in the household, when young, may impart a higher risk of developing related disorders in adulthood.

Sub-cortically, we found consistent negative associations in the dorsal striatum as a function of increasing ACEs, in both resting and CPT scans, yet these negative associations were more widespread in the CPT condition, due to the addition of the caudate. The putamen has been associated with learning and motor control, speech articulation, reward, cognitive function, and addiction (58). The caudate has been associated with coordinating decision processes, balancing external evidence, and internal preferences, along with a variety of executive processes such as goal-directed action (59, 60). Our results suggest that impaired decision-making may be associated with increasing ACEs, in part through the aberrant function of the dorsal striatum (61). Impaired decision-making is often associated with substance abuse (62, 63) and has also been associated with major depressive disorder (64, 65). Furthermore, substance abuse has been associated with decreased left putamen activation (66) and so has major depressive disorder in response to reward cues (67), both of which we found to implicate reduced function in the left putamen as either full or partial mediators but did not survive FDR correction (see **Supplementary Table S3**).

In the thalamus, we found negative associations in areas near the medial and lateral dorsal nuclei on the right and in the left pulvinar at rest. In the CPT condition, the negative associations in the left pulvinar were consistent, yet the associations in the medial dorsal nucleus were positive. The pulvinar is a component of the visual attention network, with connections to areas of the dorsal visual stream and posterior parietal cortex (68). The lateral dorsal nucleus has been associated with motivation, attention, and sensory processes with connections to the posterior cingulate and parietal cortex, both of which are positively associated with ACEs at rest, results that may be related given the projections between the posterior cingulate, lateral dorsal nucleus, and parietal cortex (69, 70). The medial dorsal nucleus has been associated with awareness, mood, motivation, sleep/wake cycles, and chronic pain and has connections with the anterior cingulate (70–72). Interestingly, we found the activation of the medial dorsal nucleus in the CPT condition to be a partial mediator of ACE associations with mood disorders, along with the pons. Our findings in the medial and lateral thalamic nuclei may indicate lower awareness, motivation, and emotional arousal at rest, which are then hyperactivated in the CPT condition as a function of increasing ACEs. Furthermore, the pons has been associated with sleep/wake cycles (73), and disruptions in circadian rhythms in mood disorders (74) and ACEs have been associated with sleep disturbances (75), suggesting that decreased function in the medial dorsal thalamus and pons may be associated with sleep disturbances, particularly in patients with mood disorders, although we did not find any mediating associations with these regions in the primary sleep disorder diagnostic group.

In the cingulate, we found positive associations in the superior anterior cingulate and posterior cingulate across both resting and CPT scans. In the CPT condition, we found positive associations in the ventral anterior cingulate and, at rest, in the middle cingulate. Each of these regions is part of the default mode network and appears to be overactive in both conditions with increasing ACEs. The anterior cingulate, along with its connections to the amygdala, ventral striatum, and orbitofrontal cortex, has been associated with motivation, context-dependent behavior, cognitive control, and conflict processing (76, 77), although we found no relationships with the amygdala. The posterior cingulate may play a role in regulating the focus of attention (78). In both substance abuse and dependence disorders, we found that the posterior cingulate was involved in mediating the relationship with ACEs, along with the superior aspect of the anterior cingulate at rest. These results suggest that there may be overactivity of these cingulate regions with increasing ACEs and may be related to behavioral phenotypes seen in substance abuse-related disorders such as lack of motivation, impaired reasoning, and altered cognitive function (79).

In the cerebellum, we found negative associations in both resting and CPT conditions. The negative associations are bilateral and consistent in spatial location in both conditions. The cerebellum has historically been associated with motor functions but more recently has received attention for its role in social cognition (80). Cerebellar activation has been associated with tasks ranging from attention, executive control, language, working memory, learning, pain, emotion, and addiction (81). Using a cerebellar atlas created from resting-state functional connectivity data from 1,000 subjects (82, 83), we overlaid the seven network atlas and found that the negative associations in the cerebellar regions overlap with the ventral attention network, which Yeo et al. describe as an aggregate of salience networks found in the literature. The ventral attention network comprises the temporoparietal junction and the ventral frontal cortex (84). The temporoparietal junction has been associated with reorienting attention to unexpected stimuli (85). In our results, we see positive associations in the temporoparietal junction area (stronger at CPT than at rest) and negative associations in the ventral frontal cortex (i.e., BA 47). These results lead us to speculate that there may be a dysfunction in stimulus-driven attention and the switching of attention from internal to exogenous stimuli as a function of increasing ACEs, which is more evident in SPECT scans acquired during an attention task but also evident, to a lesser extent, at rest.

### 4.2 Clinical manifestations and practical implications

The study’s findings indicate that ACEs are linked to altered brain function in several critical regions, including the cognitive control network, the default mode network, and areas of the dorsal striatum and cerebellum. These brain changes are associated with an increased risk of psychiatric disorders such as PTSD, anxiety disorders, substance use disorders, and depression. Clinically, this suggests that individuals with high ACE scores may present with more complex and treatment-resistant forms of these conditions due to the underlying neurobiological alterations.

Clinicians should be aware that the altered brain activity patterns observed in this study may manifest as difficulties in decision-making, emotional regulation, and cognitive control. For instance, overactivity in the medial frontal lobe and anterior cingulate cortex (parts of the cognitive control network) may lead to heightened emotional responses and difficulty in regulating emotions, which are common in PTSD and mood disorders. Similarly, decreased activity in the dorsal striatum may contribute to impaired reward processing and motivation, which are often seen in substance use disorders and depression.

To integrate these findings into clinical practice, clinicians should:

- *Screen for ACEs*: Implement routine screening for ACEs in psychiatric evaluations to identify individuals at higher risk for complex mental health issues. Tools like the ACE questionnaire used in this study can be easily administered during patient intake.
- *Personalize Treatment Plans*: Recognize that patients with high ACE scores may benefit from personalized treatment plans that address both the psychological and neurobiological aspects of their conditions. This could involve a combination of psychotherapy, such as trauma-focused cognitive behavioral therapy (CBT), and neurobiologically-informed interventions like neurofeedback or brain stimulation therapies.
- *Monitor Brain Function*: Consider using functional neuroimaging, such as SPECT scans, to monitor brain activity in patients with high ACE scores. This can provide valuable information on brain regions that may need targeted intervention and help track treatment progress.
- *Adopt a Holistic Approach*: Adopt a holistic approach to treatment that includes addressing lifestyle factors known to influence brain health. Encourage patients to engage in regular physical activity, maintain a healthy diet, and practice stress-reduction techniques such as mindfulness and meditation, which can improve overall brain function and resilience.
- *Practice Interdisciplinary Collaboration*: Collaborate with other healthcare providers, including primary care physicians, neurologists, and social workers, to create comprehensive care plans that address the multifaceted needs of patients with high ACE scores.

### 4.3 Study limitations

This study has several limitations. First, the study group consists of a large clinical sample with varying diagnoses (see **Supplementary Table S1.1**) and comorbidities, across a wide range of ages and differing sex at birth. We have tried to reduce these confounders by adding regressors to our models, but we know that linear models alone will not mitigate these effects on our results. We saw some of this reflected in the small effect sizes of the neuroimaging associations with ACEs, suggesting that a large sample is needed to produce the statistically significant associations as we have shown here. Although no patients with dementia or mild cognitive impairment were included in this sample, the cohort did include older adults and some who reported having brain trauma in their lives. It is possible that their self-report of ACEs was inaccurate as they may have undiagnosed age or trauma-related memory problems. Furthermore, a subset of patients (*N* = 588) reported taking psychiatric medications prescribed prior to visiting the Amen Clinics (see **Supplementary Table S5.1**), which could alter perfusion in these patients and increase the overall variability of the neuroimaging data. We ran a sensitivity analysis, removing these 588 patients, and found the results to be consistent with those reported in the manuscript (see **Supplementary Figures S5.2** and **S5.3**). Next, treating physicians gave each patient the DSM-based diagnoses used in this study. The physicians had access to all available data, including patient-reported symptoms and reports on large regions in the SPECT neuroimaging data that were deemed over- or underactive by trained scan readers. Although this is certainly a confounding factor, none of the physicians had access to the quantitative results shown here or made a diagnosis based solely on the SPECT scan data. In interpreting the SPECT scan associations with ACEs, we identified brain regions using a brain atlas and MNI-space coordinates of the largest (i.e., peak-level) associations after whole-brain thresholding. Some of these anatomical regions are very small (e.g., medial dorsal thalamus), and the SPECT scan resolution (~6.5 mm) was not sufficient to definitively say that such signals originate from these small regions. Additional studies should be done to validate these findings and to understand whether these effects are unique to SPECT imaging or are reproducible in related technologies such as PET and functional MRI. In addition, we attempted to interpret the neuroimaging findings with respect to the anatomical regions implicated by the maximum statistical strength and associate these with prior literature, but we acknowledge that the brain is a highly interconnected organ and there are likely more complex interactions occurring between brain regions that would require other modalities to investigate. Furthermore, we know that each DSM-based diagnosis includes a variety of symptoms that are not necessarily consistent across patients with the same diagnosis. If we assume that a patient’s symptoms are related to their brain function, we would expect these problems to increase the variability of the SPECT scans. Our results show the most consistent patterns associated with ACEs across the diagnostic groups included in the cohort and may not reflect patterns that could be seen by radiologists on individual patient scans in isolation. Despite these limitations, we believe that the results presented here show the common brain functional features associated with increasing ACEs and their relationship with the diagnoses in a heterogeneous clinical dataset, using data-driven approaches.

## 5 Conclusions

The findings from this study highlight the critical role of brain function in mediating the relationship between ACEs and psychiatric diagnoses. By understanding these neurobiological underpinnings, clinicians can better tailor interventions to address the specific needs of individuals with high ACE scores. This approach not only improves diagnostic accuracy but also enhances treatment efficacy, ultimately leading to better patient outcomes. Future research should continue to explore the neurobiological mechanisms underlying the impact of ACEs on mental health, with an emphasis on longitudinal studies, to track changes over time and the effectiveness of targeted interventions. By advancing our understanding of these complex interactions, we can develop more effective strategies to mitigate the long-term effects of childhood adversity on mental health.

## Data Availability

Due to the sensitive nature of this clinical data, it has not been posted publicly, but the anonymized data used in this study is freely available for research by request. Requests to access the datasets should be directed to dkeator@changeyourbrainfoundation.org

## Footnotes

1
https://www.fil.ion.ucl.ac.uk/spm/software/spm12/.

2
https://www.r-project.org.

3
https://mangoviewer.com/.
