# Supplementary material for: Adverse childhood experiences, brain function, and psychiatric diagnoses in a large adult clinical cohort
Source: Front Psychiatry. 2024 Oct 14;15:1401745. doi: 10.3389/fpsyt.2024.1401745 (PMC11513356; doi:10.3389/fpsyt.2024.1401745)

# *Supplementary Material*

**S1: Diagnostic Classes**

To evaluate the relationship between diagnostic classes and the risk associated with increasing reported ACEs, we classified the Amen Clinics diagnoses into classes. Using these diagnostic classes, we computed the frequency of each diagnostic class within the ACEs sample used in this study (see Table S1.1).

Table S1.1: Frequency in percent of each diagnosis in the ACEs sample with a frequency of 1.0% or greater. Each patient may have more than one diagnosis.

| Diagnostic Class | Frequency (%) |
|---|---|
| Anxiety Disorder | 70.0 |
| Depressive Disorder | 44.5 |
| Major Depression | 41.6 |
| Attention Deficit Disruptive Behavior | 38.6 |
| Attention Deficit Hyperactivity | 38.5 |
| Generalized Anxiety Disorder | 30.7 |
| Post-Traumatic Stress Disorder | 27.0 |
| Substance Abuse Disorder | 11.4 |
| Depression Not Otherwise Specified (NOS) | 9.2 |
| Bipolar Disorder | 8.9 |
| Mood Disorder NOS | 8.6 |
| Alcohol Related Disorder | 7.6 |
| Adjustment Disorder | 7.0 |
| BrainTrauma | 6.6 |
| Phobias | 5.4 |
| Obsessive Compulsive Disorder | 5.0 |
| Substance Dependence | 4.9 |

| | |
|---|---|
| Dysthymic_Disorder | 4.7 |
| Social Phobia | 4.6 |
| Nicotine Related Disorders | 2.8 |
| Primary Sleep Disorder | 2.7 |
| Pervasive Developmental Disorders | 2.7 |
| Frontal Lobe Dysfunction | 2.5 |
| Eating Disorder | 2.3 |
| Other Sleep Disorders | 2.1 |
| Eating Disorder Unspecified | 2.0 |
| Autism | 2.0 |
| Personallity_Cluster_B | 1.3 |
| Borderline Personality Disorder | 1.3 |
| Learning Disorder | 1.2 |
| Cyclothymic Disorder | 1.2 |
| Parasomnia | 1.1 |
| Dyslexia | 1.0 |

**S2: Questions by Diagnosis**

To evaluate the relationship between specific ACE questions and each diagnostic class, we constructed linear models of the form diagnostic_class = ACE_Q1 + ACE_Q2 +... + ACE_Q10 + sex + age + site for each patient where the diagnostic class indicator identified patients who had the diagnosis, compared to the others who did not. We then evaluated which of the ACE questions were significantly related to each diagnostic class at the level of $p<0.05$ FDR. In Table S2.1 we provide the resulting statistics for each diagnostic class with at least a one percent frequency.

Table S2.1: Z-scores and FDR-corrected probability of each ACEs questions in association with each diagnosis that was statistically related to increasing ACEs (DOF=7164).

| Diagnosis | Q1 Z | Q1 p(fdr) | Q2 Z | Q2 p(fdr) | Q3 Z | Q3 p(fdr) | Q4 Z | Q4 p(fdr) | Q5 Z | Q5 p(fdr) | Q6 Z | Q6 p(fdr) | Q7 Z | Q7 p(fdr) | Q8 Z | Q8 p(fdr) | Q9 Z | Q9 p(fdr) | Q10 Z | Q10 p(fdr) |
|---|---|---|---|---|---|---|---|---|---|---|---|---|---|---|---|---|---|---|---|---|
| Anxiety Disorder | 2.67 | 7.9E-02 | -0.02 | 1.0E+00 | 2.11 | 1.2E-01 | 2.33 | 1.1E-01 | 1.85 | 2.4E-01 | 1.46 | 3.8E-01 | 2.22 | 2.2E-01 | -0.85 | 7.5E-01 | 4.39 | 1.9E-04 | -0.32 | 9.5E-01 |

| | | | | | | | | | | | | | | | | | | | | |
|---|---|---|---|---|---|---|---|---|---|---|---|---|---|---|---|---|---|---|---|---|
| Depressive Disorder | 2.37 | 7.9E-02 | -0.38 | 1.0E+00 | 0.5 | 8.1E-01 | 3.32 | 1.5E-02 | -1.59 | 3.5E-01 | -1.05 | 4.2E-01 | 0.71 | 7.5E-01 | 0.13 | 9.5E-01 | 3.72 | 1.7E-03 | -0.19 | 9.5E-01 |
| Major Depression | 2.34 | 7.9E-02 | -0.64 | 8.5E-01 | -0.29 | 8.1E-01 | 3 | 3.0E-02 | -1.53 | 3.5E-01 | -1.35 | 3.8E-01 | 0.41 | 7.6E-01 | -0.19 | 9.4E-01 | 2.91 | 2.0E-02 | -0.2 | 9.5E-01 |
| Attention Deficit Disruptive Behavior | 2.4 | 7.9E-02 | 1.4 | 5.5E-01 | 2.86 | 3.5E-02 | 2.04 | 1.3E-01 | -1.99 | 2.4E-01 | 1.65 | 3.4E-01 | -2.84 | 5.0E-02 | 1.11 | 6.8E-01 | 1.21 | 4.0E-01 | 0.77 | 9.5E-01 |
| Attention Deficit hyperactivity | 2.41 | 7.9E-02 | 1.38 | 5.5E-01 | 2.89 | 3.5E-02 | 2.03 | 1.3E-01 | -1.95 | 2.4E-01 | 1.63 | 3.4E-01 | -2.91 | 5.0E-02 | 1.16 | 6.7E-01 | 1.13 | 4.1E-01 | 0.79 | 9.5E-01 |
| PTSD | 4.84 | 4.3E-05 | 2.55 | 1.8E-01 | 11.29 | 4.8E-28 | 6.08 | 4.0E-08 | 6.1 | 3.4E-08 | 2.19 | 2.3E-01 | 3.07 | 5.0E-02 | 0.25 | 9.4E-01 | 4.28 | 2.1E-04 | 0.73 | 9.5E-01 |
| Substance Abuse Disorder | 0.89 | 5.9E-01 | 0.93 | 6.8E-01 | 0.97 | 6.0E-01 | 2.22 | 1.3E-01 | -1.13 | 6.6E-01 | 0.71 | 5.8E-01 | -0.86 | 7.5E-01 | 5.02 | 8.7E-06 | -2.6 | 4.1E-02 | 1 | 9.5E-01 |
| Depression NOS | 1.3 | 4.6E-01 | 0.95 | 6.8E-01 | 1.17 | 5.0E-01 | 0.05 | 9.6E-01 | 0.41 | 8.0E-01 | 1.95 | 2.8E-01 | 0 | 1.0E+00 | 0.31 | 9.4E-01 | 4.81 | 4.9E-05 | -0.18 | 9.5E-01 |
| Bipolar Disorder | 0.81 | 6.2E-01 | -0.04 | 1.0E+00 | 2.39 | 8.7E-02 | 1.06 | 5.1E-01 | 0.47 | 8.0E-01 | -0.26 | 8.2E-01 | 0.45 | 7.6E-01 | 0.68 | 8.2E-01 | 2.31 | 6.3E-02 | 0.81 | 9.5E-01 |
| Mood Disorder NOS | 0.89 | 5.9E-01 | -0.14 | 1.0E+00 | 0.71 | 7.5E-01 | 0.09 | 9.6E-01 | -1.53 | 3.5E-01 | 1.65 | 3.4E-01 | 0.8 | 7.5E-01 | 1.24 | 6.4E-01 | 1.31 | 3.5E-01 | 0.11 | 9.5E-01 |
| Alcohol Related Disorder | -0.3 | 8.9E-01 | 0.65 | 8.5E-01 | 1.93 | 1.5E-01 | 1.4 | 3.3E-01 | 0.54 | 8.0E-01 | 0.32 | 8.1E-01 | -0.66 | 7.5E-01 | 5.49 | 1.3E-06 | -2.33 | 6.3E-02 | -1.62 | 9.5E-01 |
| Brain Trauma | 3.18 | 2.5E-02 | 1.65 | 4.9E-01 | 2.06 | 1.2E-01 | 2.9 | 3.1E-02 | -2.36 | 1.2E-01 | 3.18 | 4.9E-02 | 0.69 | 7.5E-01 | 0.82 | 7.5E-01 | 0.3 | 8.0E-01 | 0.48 | 9.5E-01 |
| Phobias | -0.15 | 9.1E-01 | 1.03 | 6.8E-01 | -1.1 | 5.2E-01 | 0.58 | 6.8E-01 | 2.62 | 7.3E-02 | -1.63 | 3.4E-01 | 0.57 | 7.5E-01 | -0.21 | 9.4E-01 | 0.96 | 5.0E-01 | 1.15 | 9.5E-01 |
| Substance dependence | 1.09 | 5.5E-01 | -0.01 | 1.0E+00 | -0.32 | 8.1E-01 | 0.48 | 7.2E-01 | 0.45 | 8.0E-01 | -0.92 | 4.5E-01 | -0.59 | 7.5E-01 | 3.62 | 3.2E-03 | -0.68 | 6.6E-01 | -0.35 | 9.5E-01 |
| Nicotine related disorders | -0.1 | 9.2E-01 | -0.24 | 1.0E+00 | -2.34 | 8.7E-02 | 2.8 | 3.4E-02 | 0.93 | 6.8E-01 | 1.23 | 3.8E-01 | 0.12 | 9.3E-01 | 2.87 | 3.4E-02 | -2.58 | 4.1E-02 | 0.39 | 9.5E-01 |
| Primary Sleep Disorder | -1.04 | 5.5E-01 | 1.58 | 4.9E-01 | -0.94 | 6.0E-01 | 1.57 | 2.9E-01 | -0.5 | 8.0E-01 | 1.19 | 3.8E-01 | 0.64 | 7.5E-01 | 0.87 | 7.5E-01 | 0.58 | 6.9E-01 | -0.63 | 9.5E-01 |
| Personality Cluster B | 1.61 | 2.9E-01 | 1.8 | 4.8E-01 | 1.42 | 3.5E-01 | 2.05 | 1.3E-01 | 0.2 | 8.7E-01 | 0.45 | 7.5E-01 | -0.46 | 7.6E-01 | -0.29 | 9.4E-01 | 1.39 | 3.2E-01 | 0.36 | 9.5E-01 |
| Borderline | 1.76 | 2.4E-01 | 1.81 | 4.8E-01 | 1.46 | 3.5E-01 | 2.14 | 1.3E-01 | 0.23 | 8.7E-01 | 0.3 | 8.1E-01 | -0.4 | 7.6E-01 | -0.47 | 8.8E-01 | 1.51 | 3.0E-01 | 0.38 | 9.5E-01 |
| Cyclothymic Disorder | 1.03 | 5.5E-01 | -1.11 | 6.8E-01 | 2.3 | 8.7E-02 | 1.61 | 2.9E-01 | -0.96 | 6.8E-01 | -1.95 | 2.8E-01 | 0.88 | 7.5E-01 | 0.54 | 8.8E-01 | 0.93 | 5.1E-01 | 1.04 | 9.5E-01 |
| Parasomnia | 1.26 | 4.6E-01 | 0.03 | 1.0E+00 | 1.41 | 3.5E-01 | -0.07 | 9.6E-01 | 3.42 | 1.0E-02 | -0.96 | 4.4E-01 | 1.13 | 6.5E-01 | 2.57 | 6.7E-02 | 0.38 | 7.7E-01 | 0.21 | 9.5E-01 |

## S3: ACEs - Brain Function Mediation

In the main manuscript, section 3.1, we showed the significant brain functional associations with increasing ACEs. In section 3.2 we related increasing ACEs to the risk of being diagnosed with specific mental health conditions. Our hypothesis is that having ACEs as a child may alter one's perception of the world, their experiences in it, and brain development/function. Such aberrant brain function, in adulthood, could be a potential mediator between ACEs and a mental health diagnosis. For a full mediation we required that the probabilities on the coefficients satisfy the following criteria: $a(p) < 0.05$, $b(p) < 0.05$, $ab(p) < 0.05$, and $c'(p) > 0.05$, indicating that the path through the brain function mediator variable is significant whereas the direct path (c') after controlling for the mediator is not. For partial mediation we required: $a(p) < 0.05$, $b(p) < 0.05$, $ab(p) <$

0.05, and allow c'(p) < 0.05 but that a(p), b(p), ab(p) are all less than (i.e. more significant) the c'(p) relationship, indicating that the path through the brain function mediator variable is more statistically significant than the direct path (c') after controlling for the mediator. Here we provide a table (Table S3.1) including the full and partial mediation results, including those that did not survive FDR multiple comparison corrections but were significant at the uncorrected level.

Table S3.1: Mediation model results by diagnostic class. The table shows the central coordinate of the ROI used to sample SPECT data (Brain Region), condition the result applies to (i.e. rest or CPT), mediation type (i.e. full or partial), t-score (1,7207), and uncorrected p-values for each path in the mediation model. Mediation results surviving FDR correction are indicated in bold.

| Diagnostic Class | Brain Region (coordinates) | Condition | Mediation Type | a(t) | a(p) | b(t) | b(p) | ab(t) | ab(p) | c'(t) | c'(p) |
|---|---|---|---|---|---|---|---|---|---|---|---|
| Substance Abuse Disorder | **Putamen L (-20,16,-4)** | **rest** | **full** | **-5.12** | **3.10E-07** | **3.04** | **2.37E-03** | **-2.58** | **9.95E-03** | **1.48** | **1.38E-01** |
| | Angular Gyrus R (42,-52,40) | rest | full | 5.41 | 6.69E-08 | -2.26 | 2.41E-02 | -2.05 | 4.01E-02 | 1.44 | 1.49E-01 |
| | Medial Frontal (0,42,-22) | rest | full | 3.19 | 1.44E-03 | -2.63 | 8.67E-03 | -1.97 | 4.89E-02 | 1.40 | 1.61E-01 |
| | **Insula L (-32,6,16)** | **cpt** | **full** | **-5.04** | **4.74E-07** | **3.53** | **4.16E-04** | **-2.85** | **4.32E-03** | **1.51** | **1.31E-01** |
| | **Caudate L (-14,16,-2)** | **cpt** | **full** | **-5.51** | **3.65E-08** | **2.86** | **4.28E-03** | **-2.51** | **1.23E-02** | **1.49** | **1.37E-01** |
| | Temporal Superior L (-58,-30,16) | cpt | full | 4.59 | 4.44E-06 | -2.65 | 8.13E-03 | -2.25 | 2.42E-02 | 1.44 | 1.49E-01 |
| | Medial Frontal (4,40,-24) | cpt | full | 3.65 | 2.60E-04 | -2.52 | 1.18E-02 | -2.02 | 4.31E-02 | 1.41 | 1.58E-01 |
| | Posterior Cingulate (0,-42,36) | cpt | full | 4.23 | 2.38E-05 | -2.32 | 2.02E-02 | -1.99 | 4.62E-02 | 1.42 | 1.56E-01 |
| Substance Dependence | **Medial Frontal (0,58,16)** | **rest** | **full** | **4.57** | **4.85E-06** | **-2.81** | **4.92E-03** | **-2.36** | **1.85E-02** | **0.52** | **6.02E-01** |
| | Parietal Superior L (-26,-60,50) | rest | full | 3.49 | 4.93E-04 | -2.99 | 2.81E-03 | -2.22 | 2.66E-02 | 0.49 | 6.22E-01 |
| | Posterior Cingulate (-4,-60,8) | rest | full | 2.95 | 3.17E-03 | -3.19 | 1.42E-03 | -2.11 | 3.47E-02 | 0.48 | 6.30E-01 |
| | Anterior Cingulate Superior (0,30,26) | rest | full | 2.50 | 1.25E-02 | -4.00 | 6.46E-05 | -2.07 | 3.82E-02 | 0.49 | 6.26E-01 |
| | Insula R (44,-22,14) | rest | full | 3.24 | 1.20E-03 | -2.69 | 7.15E-03 | -2.01 | 4.40E-02 | 0.47 | 6.36E-01 |
| | **Medial Frontal (-2,60,14)** | **cpt** | **full** | **5.05** | **4.56E-07** | **-2.49** | **1.29E-02** | **-2.20** | **2.80E-02** | **0.52** | **6.05E-01** |
| | **Medial Frontal L (-16,44,10)** | **cpt** | **full** | **-3.64** | **2.70E-04** | **2.80** | **5.07E-03** | **-2.17** | **3.00E-02** | **0.49** | **6.24E-01** |
| | Temporal Superior L (-58,-30,16) | cpt | full | 4.59 | 4.44E-06 | -2.51 | 1.20E-02 | -2.17 | 3.03E-02 | 0.51 | 6.13E-01 |
| | Parahippocampal L (-20,-40,-12) | cpt | full | 3.49 | 4.79E-04 | 2.74 | 6.15E-03 | 2.10 | 3.54E-02 | 0.26 | 7.97E-01 |
| | Anterior Cingulate Superior L (-2,34,22) | cpt | full | 2.76 | 5.80E-03 | -3.03 | 2.47E-03 | -1.98 | 4.76E-02 | 0.47 | 6.39E-01 |
| Alcohol Related Disorder | **Parietal Superior L (-26,-60,50)** | **rest** | **partial** | **3.49** | **4.93E-04** | **-3.96** | **7.60E-05** | **-2.57** | **1.02E-02** | **2.25** | **2.43E-02** |

| | | | | | | | | | | | |
|---|---|---|---|---|---|---|---|---|---|---|---|
| | **Parahippocampal R (18,-34,-6)** | **cpt** | **full** | **4.52** | **6.24E-06** | **2.40** | **1.62E-02** | **2.08** | **3.73E-02** | **1.96** | **5.01E-02** |
| | **Insula L (-32,6,16)** | **cpt** | **partial** | **-5.04** | **4.74E-07** | **2.77** | **5.61E-03** | **-2.39** | **1.68E-02** | **2.25** | **2.44E-02** |
| | Medial Frontal L (-16,44,10) | cpt | partial | -3.64 | 2.70E-04 | 3.05 | 2.28E-03 | -2.29 | 2.21E-02 | 2.22 | 2.65E-02 |
| Nicotine Related Disorder | **Pons (2,-32,-38)** | **cpt** | **full** | **-5.14** | **2.86E-07** | **-2.24** | **2.52E-02** | **2.02** | **4.34E-02** | **0.51** | **6.10E-01** |
| Cyclothymic Disorder | Parietal Inferior R (40,-54,42) | rest | full | 5.59 | 2.42E-08 | 2.16 | 3.08E-02 | 1.99 | 4.70E-02 | 1.79 | 7.43E-02 |
| Mood Disorder NOS | **Pons (6,-28,-32)** | **rest** | **partial** | **-4.82** | **1.47E-06** | **3.35** | **8.13E-04** | **-2.71** | **6.72E-03** | **2.45** | **1.44E-02** |
| | **Parahippocampal L (-20,-40,-12)** | **cpt** | **partial** | **3.49** | **4.79E-04** | **-6.26** | **3.96E-10** | **-3.02** | **2.52E-03** | **2.52** | **1.17E-02** |
| | Parahippocampal R (18,-34,-6) | cpt | partial | 4.52 | 6.24E-06 | -3.52 | 4.41E-04 | -2.73 | 6.27E-03 | 2.45 | 1.44E-02 |
| | **Thalamus Medial Dorsal (0,-20,-2)** | **cpt** | **partial** | **4.89** | **1.04E-06** | **3.36** | **7.85E-04** | **2.73** | **6.34E-03** | **2.07** | **3.89E-02** |
| Major Depression | Putamen L (-28,10,-8) | cpt | partial | -6.15 | 8.29E-10 | -5.31 | 1.12E-07 | 3.99 | 6.70E-05 | 3.45 | 5.62E-04 |

S4: SPECT CPT – Rest Associations with ACEs

In the main manuscript, section 3.1, we showed the significant brain functional associations with increasing ACEs in the CPT and rest scans. Here we evaluate the difference images (CPT – rest) and associate the differences with the ACE total score. The clusters of significant voxels were evaluated using false discovery rate (FDR) multiple comparison corrections ($t(1,7268)=2.45$, $p<0.05$ FDR). The results are shown in Figure S4.1 and yielded only positive associations in the CPT condition over the rest condition with increasing ACEs. These results suggest that having more ACEs results in greater activation during sustained attention in regions of the frontal lobe, insula, temporal lobe, anterior, middle, and posterior cingulate, inferior parietal lobe, cerebellum, occipital lobe, caudate, and thalamus.

Figure S4.1: CPT – Rest difference associations with increasing ACE score ($t(1,7268)=2.45$; $p<0.05$ FDR corrected). Areas of red/yellow show regions of higher perfusion with increasing ACE score.

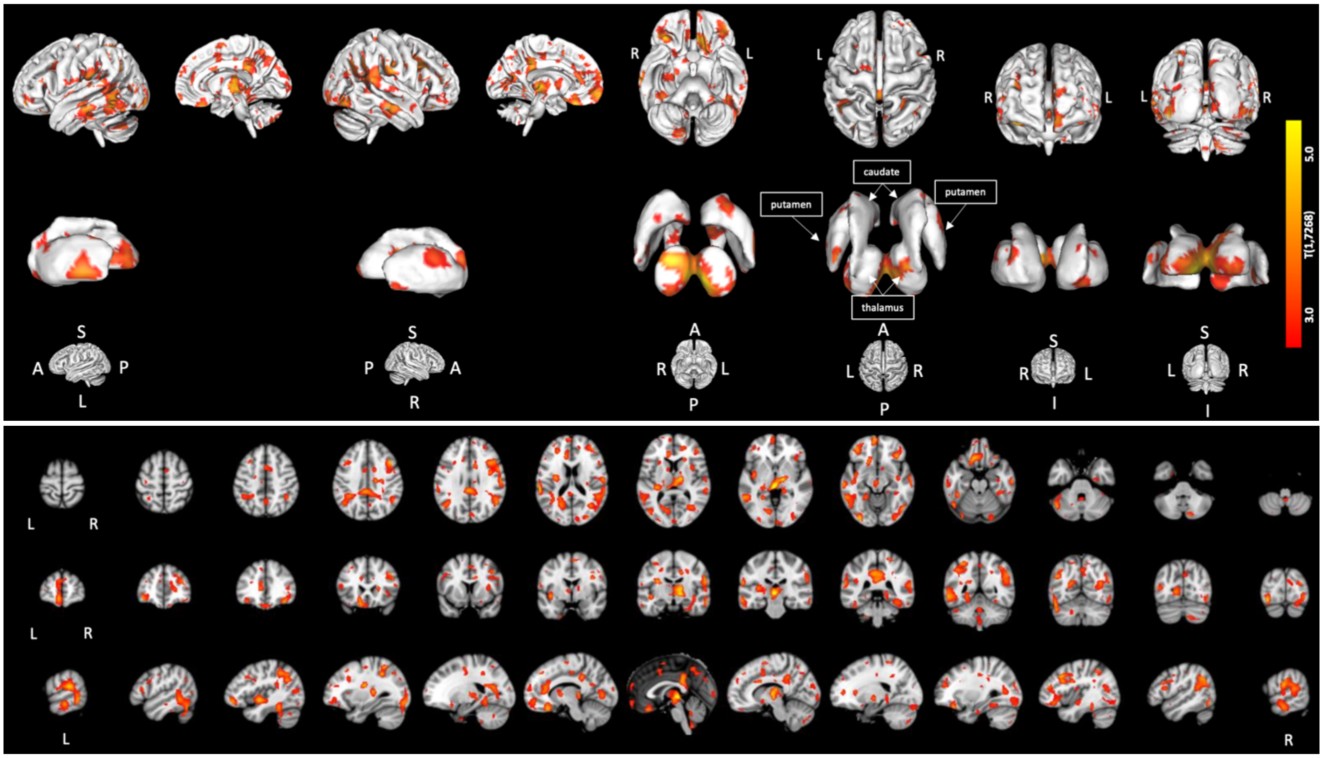

S5: Psychiatric Medications and Sensitivity Analysis

The clinical dataset used in this study consisted of 7275 patients evaluated across the eleven Amen Clinics Inc. (ACI) mental health facilities. A subset of these patients reported currently taking psychiatric medications upon intake (N=588). Table S5.1 lists the types and frequencies of psychiatric mediations within the sample used in the manuscript. In Figures S5.2 and S5.3 we show the results from a sensitivity analysis after removing the patients who reported taking psychiatric medications at intake resulting in a sample consisting of 6687 patients. The results are consistent with those reported in the paper.

Table S5.1: Psychiatric Medication Frequencies. This table shows the medication type and frequency in percent amongst the study cohort (N=7275) who reported taking psychiatric medications.

| Medication | Frequency (% sample) |
|---|---:|
| Abilify (aripiprazole) | 1.3E+00 |
| Ativan (lorazepam) | 1.4E-02 |
| Adderall | 3.5E+00 |
| Adzenys XR-ODT | 2.7E-02 |
| Alprazolam | 8.0E-01 |
| Zoloft (sertraline) | 1.4E-02 |
| Amitriptyline | 1.4E-02 |
| Amitriptyline | 1.4E-02 |
| Fluvoxamine | 1.4E-02 |
| Aripiprazole | 1.9E-01 |
| Aricept (donepezil) | 5.5E-02 |
| Ativan (lorazepam) | 7.4E-01 |
| Atomoxetine | 2.7E-02 |
| Benztropine | 1.5E-01 |
| Brintellix (vortioxetine) | 2.7E-02 |
| Bromazepam | 1.4E-02 |
| Bupropion | 1.2E+00 |
| Buspirone | 1.4E-02 |
| Cabergoline | 1.4E-02 |
| Campral | 1.4E-02 |
| Carbamazepine | 8.2E-02 |
| Carbidopa-Levodopa | 2.7E-02 |
| Citalopram | 4.4E-01 |
| Clomipramine | 4.1E-02 |
| Clonazepam | 1.4E+00 |
| Clonidine | 3.7E-01 |
| Clozapine | 8.2E-02 |
| Cymbalta (duloxetine) | 1.1E+00 |

| | |
|---|---|
| Daytrana | 2.7E-02 |
| Deplin | 5.5E-02 |
| Desipramine | 1.4E-02 |
| Desvenlafaxine | 1.8E-01 |
| Diazepam | 1.8E-01 |
| Disulfiram | 2.7E-02 |
| Donepezil | 3.4E-01 |
| Effexor (venlafaxine) | 6.7E-01 |
| Elavil (amitriptyline) | 4.1E-02 |
| Eletriptan | 1.4E-02 |
| Emgality | 1.2E-01 |
| Escitalopram | 7.7E-01 |
| Fluoxetine | 1.4E-02 |
| Fluvoxamine | 2.1E-01 |
| Geodon (ziprasidone) | 1.1E-01 |
| Glimepiride | 2.7E-02 |
| Glipizide | 5.5E-02 |
| Guanfacine | 2.2E-01 |
| Haldol (haloperidol) | 9.6E-02 |
| Hydroxyzine pamoate | 2.2E-01 |
| Imipramine hydrochloride | 2.7E-02 |
| Invega (paliperidone) | 2.3E-01 |
| Intuniv (guanfacine) | 4.1E-02 |
| Keppra (levetiracetam) | 1.6E-01 |
| Ketamine | 9.6E-02 |
| Klonopin (clonazepam) | 7.4E-01 |
| Lamictal (lamotrigine) | 9.5E-01 |
| Latuda (lurasidone) | 3.6E-01 |
| Lexapro (escitalopram) | 2.3E+00 |
| Liothyronine | 3.2E-01 |
| Lithium | 1.0E+00 |
| Lithobid | 1.4E-02 |
| Lorazepam | 1.0E+00 |
| Luvox (fluvoxamine) | 1.1E-01 |
| Mirtazapine | 5.8E-01 |
| Mirtazapine | 8.2E-02 |
| Mydayis | 6.9E-02 |
| Naltrexone | 4.7E-01 |
| Namenda (memantine) | 4.1E-02 |
| Nortriptyline | 1.5E-01 |
| Nuvigil (armodafinil) | 4.1E-02 |

| | |
|---|---|
| Olanzapine | 4.7E-01 |
| Prozac (fluoxetine) | 1.4E+00 |
| Qelbree (viloxazine) | 1.4E-02 |
| Quetiapine | 4.5E-01 |
| Qsymia | 4.1E-02 |
| Risperidone | 5.5E-01 |
| Ritalin (methylphenidate) | 3.2E-01 |
| Rizatriptan | 1.4E-02 |
| Rivastigmine | 1.4E-02 |
| Rivotril (clonazepam) | 4.1E-02 |
| Ropinirole | 6.9E-02 |
| Saphris (asenapine) | 2.7E-02 |
| Seroquel (quetiapine) | 1.2E+00 |
| Seroxat (paroxetine) | 1.4E-02 |
| Sertraline | 1.3E+00 |
| Silenor (doxepin) | 1.4E-02 |
| Sinogan | 1.4E-02 |
| Sonata (zaleplon) | 2.7E-02 |
| Spravato (ketamine) | 1.4E-02 |
| Strattera (atomoxetine) | 4.3E-01 |
| Tegretol (carbamazepine) | 5.5E-02 |
| Temazepam | 1.1E-01 |
| Topiramate | 1.8E-01 |
| Trazodone | 2.5E+00 |
| Tramadol | 2.1E-01 |
| Tremfaya (guselkumab) | 2.7E-02 |
| Trintellix (vortioxetine) | 2.6E-01 |
| Trokendi XR (topiramate) | 1.4E-02 |
| Unisom (doxylamine) | 1.4E-02 |
| Valium (diazepam) | 1.8E-01 |
| Valproic acid | 2.7E-02 |
| Venlafaxine | 5.1E-01 |
| Vortioxetine | 1.4E-02 |
| Vraylar (cariprazine) | 3.0E-01 |
| Vyvanse (lisdexamfetamine) | 1.6E+00 |
| Wellbutrin (bupropion) | 2.5E+00 |
| Xanax (alprazolam) | 1.4E+00 |
| Zaleplon | 2.7E-02 |
| Zanaflex (tizanidine) | 2.7E-02 |
| Ziprasidone | 4.1E-02 |
| Zolpidem | 3.3E-01 |

| | |
|---|---|
| Zyprexa (olanzapine) | 7.0E-01 |

Figure S5.2: Cortical and sub-cortical surfaces showing statistically-significant (t(1,6679)=2.64; p<0.05 FDR corrected) associations with increasing ACE score. Areas of red/yellow show regions of higher perfusion with increasing ACE score. Areas of blue/green show regions of lower perfusion with increasing ACE score.

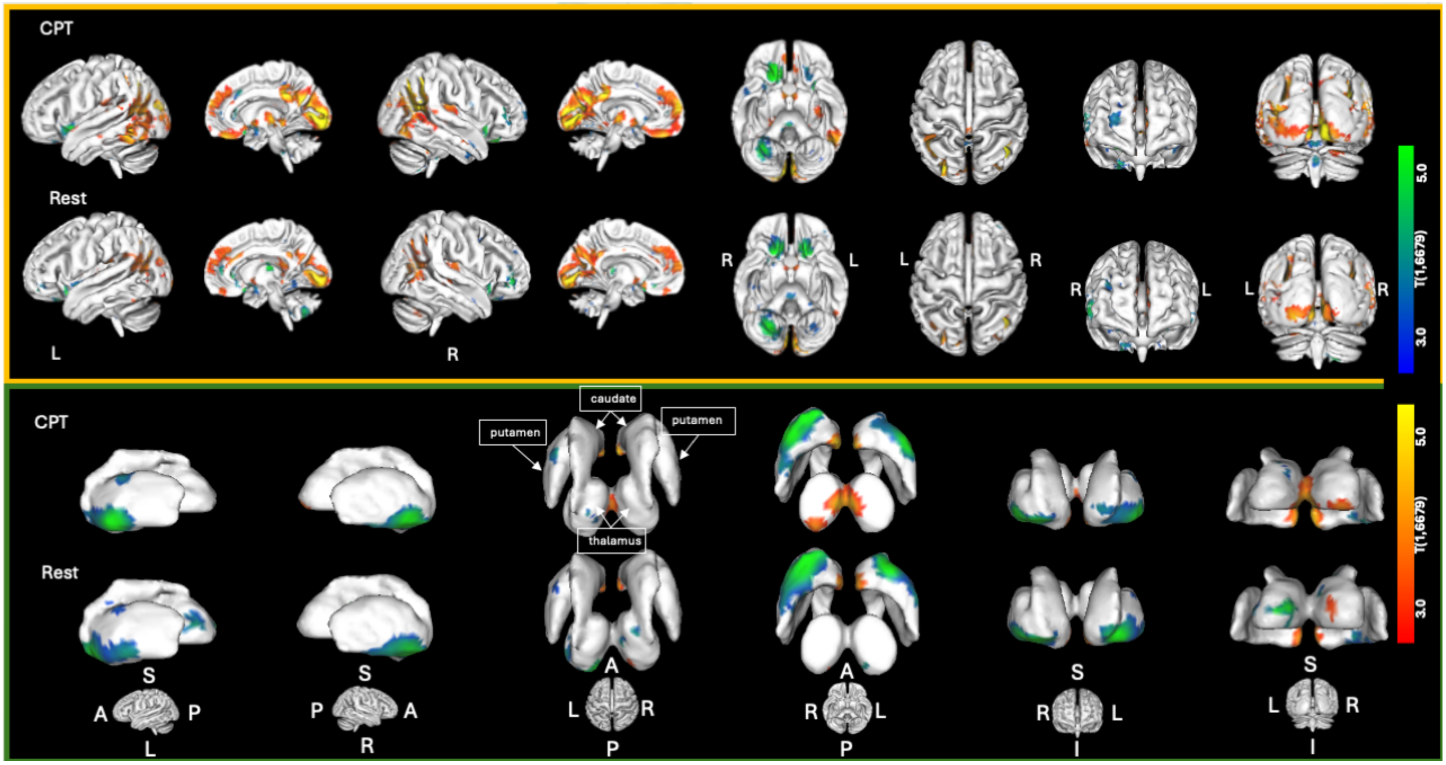

Figure S5.3: Axial, sagittal, and coronal slices showing statistically-significant (t(1,6679)=2.64; p<0.05 FDR corrected) associations with increasing ACE score. Areas of red/yellow show regions of higher perfusion with increasing ACE score. Areas of blue/green show regions of lower perfusion with increasing ACE score.

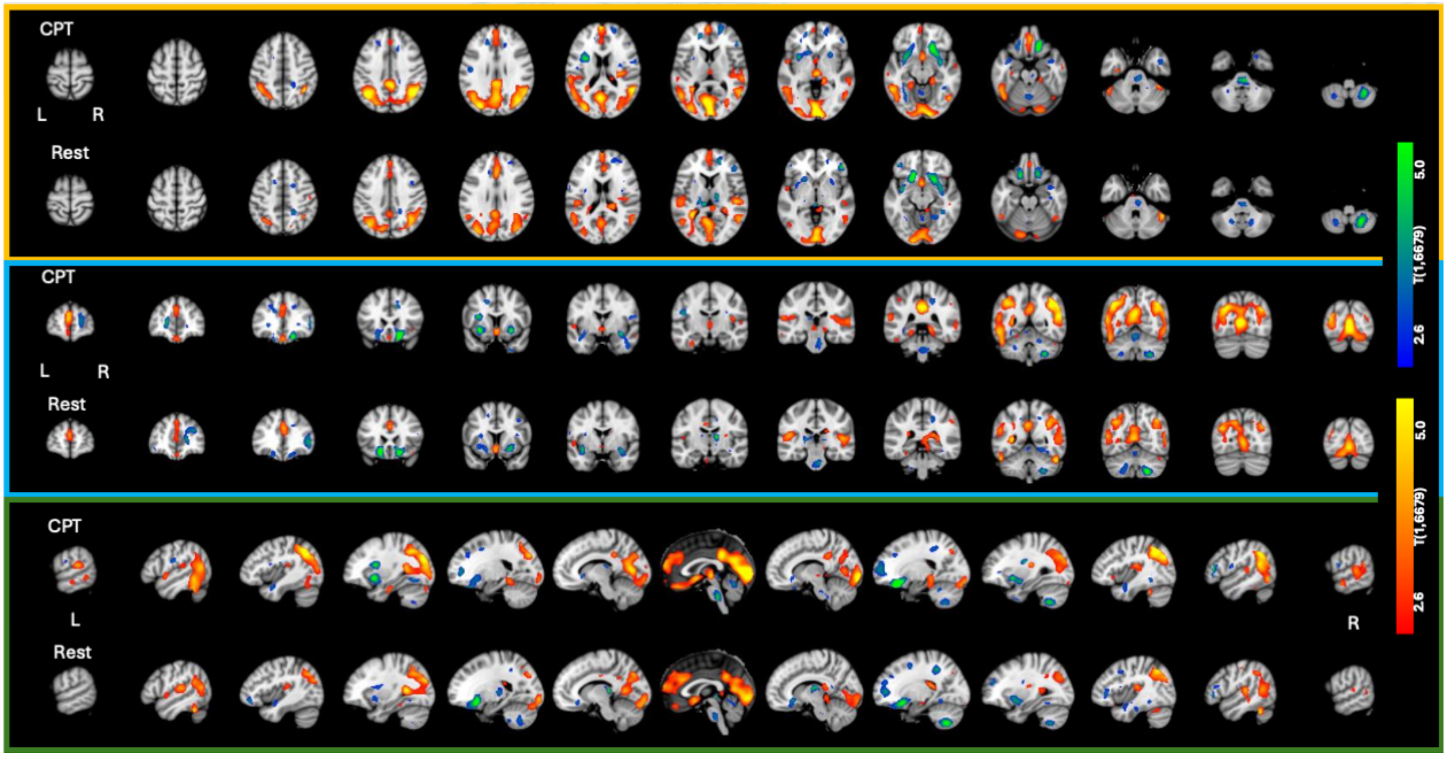

Supplement: Supplementary file 1 [file DataSheet1.pdf]
